# Genetic diversity and natural selection footprints of the glycine amidinotransferase gene in various human populations

**DOI:** 10.1038/srep18755

**Published:** 2016-01-05

**Authors:** Asifullah Khan, Lei Tian, Chao Zhang, Kai Yuan, Shuhua Xu

**Affiliations:** 1 Chinese Academy of Sciences (CAS) Key Laboratory of Computational Biology, Max Planck Independent Research Group on Population Genomics, CAS-MPG Partner Institute for Computational Biology (PICB), Shanghai Institutes for Biological Sciences, Chinese academy of Sciences, Shanghai 200031, China; 2 Department of Biochemistry, Abdul Wali Khan University Mardan (AWKUM), Mardan, Khyber Pakhthunkhwa, Pakistan; 3 School of Life Science and Technology, ShanghaiTech University, Shanghai 200031, China; 4 Collaborative Innovation Center of Genetics and Development, Shanghai 200438, China

## Abstract

The glycine amidinotransferase gene (*GATM*) plays a vital role in energy metabolism in muscle tissues and is associated with multiple clinically important phenotypes. However, the genetic diversity of the *GATM* gene remains poorly understood within and between human populations. Here we analyzed the 1,000 Genomes Project data through population genetics approaches and observed significant genetic diversity across the *GATM* gene among various continental human populations. We observed considerable variations in *GATM* allele frequencies and haplotype composition among different populations. Substantial genetic differences were observed between East Asian and European populations (*F*_*ST*_ = 0.56). In addition, the frequency of a distinct major *GATM* haplotype in these groups was congruent with population-wide diversity at this locus. Furthermore, we identified *GATM* as the top differentiated gene compared to the other statin drug response-associated genes. Composite multiple analyses identified signatures of positive selection at the *GATM* locus, which was estimated to have occurred around 850 generations ago in European populations. As *GATM* catalyzes the key step of creatine biosynthesis involved in energy metabolism, we speculate that the European prehistorical demographic transition from hunter-gatherer to farming cultures was the driving force of selection that fulfilled creatine-based metabolic requirement of the populations.

The phosphocreatine (PCr)/creatine (Cr) system acts as a rapidly available buffer for phosphate-bound energy storage and transmission in organs that demand high and fluctuating energy. These buffer system components (i.e., PCr and Cr) are highly abundant in skeletal muscles at a concentration range of 20-40 Mm^1^. The glycine amidinotransferase gene (*GATM*) (chromosome 15q15.3) encodes a mitochondrial enzyme, L-arginine:glycineamidinotransferase (AGAT, EC 2.1.4.1) that catalyzes the first critical step of indigenous creatine biosynthesis by converting arginine and glycine to ornithine and guanidinoacetate (GAA). The *GATM* gene is 41,203 bp in size, and mutations in this gene cause hereditary Cr deficiency syndromes (OMIM 602360),which are further characterized by severe mental retardation, speech delay, epilepsy, autism, and hypotonia^2^,^3^. Elevated *GATM* expression and creatine synthesis in the myocardium have been observed in heart failure patients^4^. Some common variants in the *GATM* locus have also been reported to be significantly associated with chronic kidney diseases relating renal function^5^,^6^,^7^. In addition, some expression quantitative trait loci (eQTLs) of the *GATM* are significantly associated with statin-induced (i.e., anti-hypercholesterolemia drug) myopathy^8^. Recent studies have also explored the role of blood creatine levels in attenuating gluconeogenesis, cholesterol levels, and diet-induced obesity^9^.

Human populations have encountered substantial environmental challenges as they colonized various parts of the world. Local adaptations to various environments have largely promoted genetic diversity, which is illustrated by their phenotypic differentiation. The availability of whole genome data from continental populations around the world has provided excellent opportunities to identify genetic diversity or selection signatures across various loci. Because the *GATM* gene has been implicated in several chronic diseases and drug response-relevant phenotypes among different human populations, we screened for continental-wide diversity across this gene. Analysis of the 1,000 Genomes Project data (http://www.1000genomes.org) has indicated significant continental-wide genetic diversity among human populations at the *GATM* locus. Furthermore, statistical analysis has revealed that the *GATM* gene in European populations underwent positive selection.

## Results

### Genetic differentiation of the *GATM* locus among various human populations

**Figure 1:**
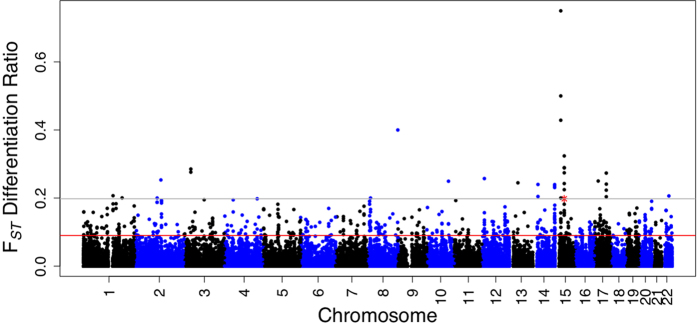
Manhattan plot using the *F*_*ST*_ differentiation ratio across all genome-wide genes, including the *GATM* gene. The plot showing the *F*_*ST*_ differentiation ratio (see Materials and Methods) in all genome-wide genes, including the *GATM* gene along with 15-kb of the flanking (upstream and downstream) regions. The analysis ranked the *GATM* gene as 33^rd^ (from a total of 54,740) highly differentiating gene, with a *F*_*ST*_ differentiation ratio = 0.20 (148/748), as represented by gray lines, whereas the red line indicates the top 1% threshold of the whole-genome level *F*_*ST*_ differentiation ratio. The *GATM* gene is indicated by an asterisk.

**Figure 2:**
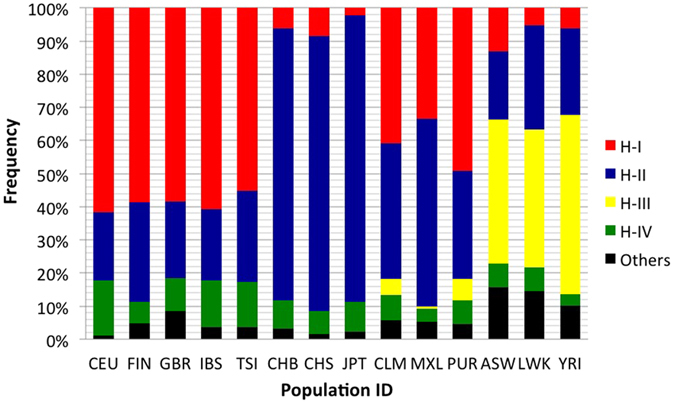
A comparison of *GATM* haplotype distribution among various populations. Three GWAS and three non-synonymous SNPs and 20 SNPs with top 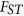 values were selected for haplotype construction and comparison.

The statistical methods developed for the calculation of population genetic differentiation are powerful tools for the identification of population-wide diverse loci in the human genome that have undergone natural selection. We employed a basic statistic to understand global genetic diversity across the *GATM* gene in 1,092 unrelated individuals from four continental regions, including Europe, East Asia, Africa, and America, from the 1,000 Genomes Project phase I. The *F*_*ST*_ differentiation ratio (See Materials and Methods section) calculated for the *GATM* locus compared to all the other 54,740 genome-wide SNPs encompassing genes ranked *GATM* as the 33^rd^ most highly differentiated gene (Fig. 1; Supplementary File 1). We calculated the pairwise 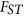 values of this gene among populations and found extreme differences between that of East Asians and Europeans, with the *F*_*ST*(CEU_JPT)_ =_ _0.56; whereas populations within these continental groups showed a high level of similarity (Supplementary Fig. S1). After observing significant differences in allele frequency of the *GATM* gene among populations, we subsequently performed haplotype analysis of each of the 14 population samples, and their abundance distributions are shown in Fig. 2. A total of four major haplotypes were detected in African ancestry populations, whereas East Asian and European groups showed three major haplotypes (Fig. 2). Congruent to the observed pairwise 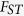 pattern, distinct haplotype diversity at the *GATM* locus was detected among population groups at the continental level. The Europeans and East Asians showed marked dichotomy with respect to the presence of major haplotypes in these groups (Fig. 2; Table 1). Haplotype-I was predominant among Europeans (average frequency: 59%), followed by the American groups consisting of European ancestry populations (i.e., CLM, MXL, and PUR; average frequency: 41.1%). However, the average frequency of haplotype-I in East Asian (CHS, CHB, and JPT) groups was 5.6%, and instead, haplotype-II was determined to be the predominant haplotype (average frequency: 83.9%). African ancestry populations comprised a distinct major haplotype (i.e., haplotype-III; average frequency: 46.4%), suggesting that it was an ancestral haplotype. Furthermore, besides Africans, this haplotype was found among American continental groups at a low frequency (i.e., average: 4%). The predominant haplotypes among Europeans and East Asians, i.e., haplotype-I and haplotype-II, were observed at an average frequency of 8.2% & 26%, respectively, among African ancestry populations.

### Comparative analysis of the *GATM* gene and other statin response-associated genes

Statins are anti-hypercholesterolemia drugs prescribed across the globe for lowering lipoprotein (LDL) concentrations. Several *GATM* eQTLs have been reported to be in association with statin-induced myopathy^8^. In a separate analysis, we applied our 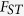 differentiation ratio (See Methods) calculation to the *GATM* and 68 other genes involved in statin drug actions and response, as collected from a PubMed literature study. Analysis showed that the *GATM* gene had the highest diversity (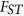 differentiation ratio >0.09) compared to the other statin response-associated genes (Supplementary Fig. S2). We also performed a population-wide analysis of several functional *GATM* eQTLs that have been previously reported to be in association with altered statin responses^8^. Among these, the two SNPs eQTLs, i.e., rs1719247 and rs1346268, showed a population-wide *F*_*ST*_ > 0.23 (i.e., 1% of the whole genome) among the 14 populations mentioned in the 1,000 Genomes Project, and their derived allele frequencies were relatively different from those of the East Asian and European population groups (Supplementary Fig. S3).

### Signatures of positive selection and linkage disequilibrium (LD) patterns at the *GATM* locus

**Figure 3:**
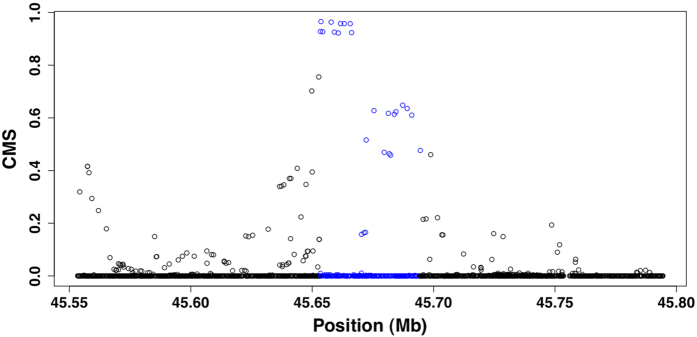
The CMS selection signal across the *GATM* locus in the CEU population. The CMS value (combined *lnRsb*, Δ*DAF*, and *F*_*ST*_) around the *GATM* gene is indicated by the blue font.

**Figure 4:**
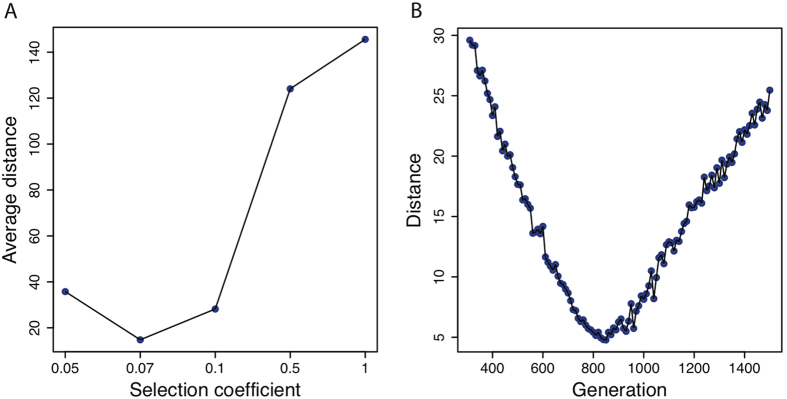
Estimation of time for natural selection. (**A**) The average distance between real and simulated data with selection coefficients of 0.05, 0.1, 0.5, and 1. (**B**) The distance between real and simulated data with different selection ages and a fixed selection coefficient of 0.07.

To investigate the potential influence of natural selection on the genetic diversity of the *GATM* gene, we applied a modified composite of multiple signals (CMS)^10^ method for the phase I dataset of the 1,000 Genomes Project, which consisted of Europeans (CEU, N = 85), Africans (YRI, N = 88), and Asians (CHB+JPT, N = 186). We integrated *lnRsb*^11^ (for the long haplotype), Δ*DAF*^10^ (for high frequency-derived alleles), and *F*_*ST*_ 12 (for highly differentiated alleles) to gain combined CMS scores at each SNP of the entire *GATM* locus (see Methods; Supplementary File 2). Both individual tests and combined CMS showed consistent signals of selection across the *GATM* gene in the CEU population, indicating the occurrence of positive selection at this locus in European populations (Fig. 3). The *lnRsb* result was shown to be more significant in these combined CMS results (Supplementary Fig. S4), indicating a fixed or nearly fixed sweep across the *GATM* locus^11^. The iHS method showed no selection signals across the *GATM* region as the sweep seemed to have reached a high frequency or fixation^13^. Moreover, we found a recombination hotspot located at 108,710 bp upstream of the *GATM* locus that may be responsible for the reduced power of iHS selection signals (Supplementary Fig. S5). Simulation-based analysis revealed an estimated selection time of approximately 17,000 years, 20 years per generation, and a selection coefficient of 0.07 (Fig. 4). To further confirm natural selection signals, linkage disequilibrium (LD) analysis was performed for various continental human populations across common SNPs covering the *GATM* gene in the 1,000 Genomes Project data. The analysis revealed a strong LD pattern for CEU populations compared to that observed in CHB and YRI populations, with a high CMS score containing SNPs (Supplementary Fig. S6). This high LD in CEU populations caused significant *lnRsb* signals^11^ during our combined CMS analysis across the *GATM* locus. The higher LD indicated lower diversity and represented selection signal at the *GATM* locus in the CEU population.

## Discussion

Modern human populations experienced a series of migrations with founder effect and subsequent population expansion^14^. During this process, distinct demographic events, surrounding climatic challenges, and food habits have resulted in favorable alleles among human populations compared to neutral loci. Estimation and analysis of such population-wide genetic structure and diversity are important for both evolutionary and medical studies^15^. The present study investigated the genetic diversity in the *GATM* gene among various human populations from four continental regions (Africa, Europe, Asia, and America). The elevated nucleotide diversity of the *GATM* gene compared to that observed in the entire genome revealed significant continental-wide population differences at the *GATM* locus, especially between East Asians and Europeans. Significant genetic diversity in haplotype diversity and LD patterns was also observed among these populations. The combined CMS results revealed positive selection across this locus in European populations. According to the ancient demography of Europeans, an important prehistorical event involving European communities was the transition from hunter-gatherer to farming cultures^16^. This Mesolithic to Neolithic transition from foraging to agricultural lifestyle was assumed to have occurred around 8,500 BC^17^. Such social and cultural transition could be associated with changes in dietary, as well as daily physiological activities of European populations. Farming was assumed to be more laborious compared to hunting. Weed evidences in southern and northern Europe suggest that early farmers invested extensive labor in the maintenance of long-established cultivation^18^. Because the meat diet is highly enriched with creatine, especially the uncooked raw meat^19^, hence we assume that hunter-gatherer individuals would have acquired sufficient creatine directly from their daily meat diet. However, the shift towards farming and cereal diets resulted in a significantly higher rate of indigenous creatine synthesis to fulfill the energy requirement for daily laborious farming. Based on this scenario, we hypothesized that the *GATM* gene might have undergone selection during the European transition from hunter-gatherer towards early farming culture. Although our estimated selection time analysis assumed more ancient *GATM* selection, which was incongruent to the timing proposed for European demographic shift towards agricultural society, it was difficult to accurately perform selection time estimation from thecurrent data.

We observed substantial genetic divergence at the *GATM* locus based on its haplotype composition among different populations. The European predominant haplotype (i.e., haplotype-I; average allele frequency: 59%) was distinct from that of East Asians’ (i.e., haplotype-II; average allele frequency: 83.8%). These considerable population variations in haplotype composition rendered it difficult to accurately predict the existing haplotype from tagged SNPs^20^. As the *GATM* gene has been associated with several important biomedical and drug relevant phenotypes^5^,^6^,^7^,^8^, the population-wide differences at this locus indicate that caution should be exercised in future association tests to eliminate spurious findings.

*GATM*-deficient mice exhibit decreased fat deposition as well as reduced cholesterol levels^9^; hence, we speculated that the genetic diversity at this locus might be associated with this relevant phenotype heterogeneity across populations. Previous studies have reported a low obesity tendency and blood cholesterol level in East Asian adults, including Japanese and Chinese populations, compared to Europeans and Americans^21^,^22^,^23^. Although obesity and blood cholesterol levels are somehow relevant to dietary intake and lifestyle, genetic and ethnic factors may also influence the expression of these phenotypes^24^,^25^,^26^. The significant genetic heterogeneity and differentiation frequency pattern of eQTLs between East Asian and European populations (Supplementary Fig. 3) at the *GATM* locus might be contributory genetic factors in this scenario. The minor allele of the *GATM cis*-eQTL (i.e., rs9806699) has been reported in association with reduced *GATM* expression in Europeans-Americans population^8^. This allele occurred at a significantly high frequency among East Asian groups (i.e. average allele frequency in CHB, CHS, and JPT = 0.75) compared to Europeans (i.e. average allele frequency in CEU, GBR, FIN and IBS = 0.29). Locus *F*_*ST*_ between CEU and JPT for rs9806699 eQTL was 0.41, with an empirical *P* value = 3.6e^−3^. The predominance of this allele in East Asian populations may contribute to the low incidence of statin-induced myopathy in East Asians compared to Europeans, as revealed in epidemiological studies^21^,^22^,^23^.

In conclusion, combined CMS statistical analysis of whole-genome sequence data from the 1,000 Genomes Project has determined that ancient fixed selection occurred in the *GATM* locus of Europeans. This selection event has resulted in an alteration in the requirement for creatine biosynthesis for energy metabolism during the prehistorical transition from foraging toward farming culture among Europeans. We also conducted an in-depth characterization of the genetic variation and haplotype structure involving the *GATM* gene among various human populations. We assumed that the significant genetic diversity at this gene locus might account for the epidemiological differences in the predisposition of creatine-associated biomedical consequences and relevant drug responses. In addition, this information provides useful resources for the design and development of epidemiological and/or anthropological studies involving the *GATM* gene.

## Materials and Methods

### Data Retrieval

The genomic data of a total of 1,092 unrelated individuals from the 1,000 Genomes Project were directly downloaded from the website (http://www.1000genomes.org). These individuals belonged to 14 populations from sub-Saharan Africa, East Asia, Europe, and the Americas. The Sub-Saharan Africans included Yoruba in Ibadan (YRI) in Nigeria; Luhya in Webuye (LWK) Kenya, and African ancestry people from Southwest United States (ASW). The European groups included residents from Northern and Western European ancestry (CEU), Toscani in Italy (TSI), British in England and Scotland (GBR), Finnish in Finland (FIN), and Iberians in Spain (IBS). The East Asians included Han Chinese in Beijing (CHB) China, Southern Han Chinese (CHS) in China, and Japanese in Tokyo (JPT), Japan. The American groups comprised Mexican ancestry individuals in Los Angeles, California (MXL); Colombians in Medellin, Colombia (CLM); and Puerto Ricans in Puerto Rico (PUR). The genetic variant datasets files (vcf format) released by the 1,000 Genomes project phase I were processed to acquire only the SNP genotype, while the rest of variants including INDELs and SVs were discarded. Total 36,820,992 SNPs from each sample of all fourteen population groups were selected for downstream analysis.

### Analysis of genetic diversity

Differences in allele frequencies among various populations were measured as unbiased *F*_*ST*_ statistics^12^. The top 1% of the whole genome locus *F*_*ST*_ was 0.23. The *F*_*ST*_ differentiation ratio was calculated for the estimation of the strength of genetic diversity at a specific gene compared to the whole-genome background. This equation comprised of


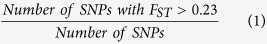


In above equation 1, the SNPs within the gene and its regulatory regions were considered. The *F*_*ST*_ differentiation ratio was compared to the empirical distribution of the *F*_*ST*_ differentiation ratios of all genes. To identify haplotype differences among populations, we constructed a haplotype that was based on 25 SNPs, i.e., top 20 *F*_*ST*_ scores containing SNPs, 3 GWAS SNPs, and 3 missense SNPs (one missense SNP was also at the top 20 *F*_*ST*_ SNPs). The haplotype frequencies were then separately calculated for each population.

### Detection of positive selection

To identify the signals underlying positive selection, the combined CMS method^10^ was implemented. Data from three continental groups provided by the 1,000 Genomes Project phase I were used: Europeans (CEU, 85 individuals), Africans (YRI, 88 individuals), and Asians (186 CHB+JPT individuals). Over 25 million SNPs in NCBI Build 37 (hg19) coordinates were analyzed. *lnRsb* was implemented in the CMS instead of XP-EHH, and iHS and ΔiHH were not integrated as they both reduced the power in cases where sweeps had reached a high frequency, fixation, or high recombination rate^10^.Δ*DAF* analysis was performed according to Grossman *et al.* and the mean values of the CEU *vs.* CHB/JPT and CEU *vs.* YRI comparisons were used to calculate the CMS score. In the case of *lnRsb*, the more significant population in these comparisons was integrated into the CMS. Unlike the study conducted by Grossman *et al.*^10^ the genome-wide empirical p-value was used instead of simulation to avoid unknown bias that could be caused by demography. The CMS score was calculated as follows:


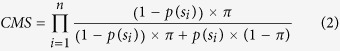


where; *p*(*s*_*i*_) is the empirical p-value of the *i*^th^ test. We assumed that 1% of the genome was under positive selection (*π* = 0.01).

### Estimation of time for natural selection

The SNP rs1153857 (i.e., containing the highest *lnRsb* score within the *GATM* gene) was selected as core SNP and an estimated 181 kb around this core SNP was assumed to have undergone natural selection (i.e., from position 45,767,079 to 45,585,610 bp), with an EHH value >0.25 and at a genetic distance of 0.055 cM. Simulation analysis was then performed to estimate the selection time of the above selected region in Europeans using the msms software^27^. We set the mutation rate as 10e^−8^ and the effective population size of Europeans as 20,000, and generated 85*2 haplotypes in each simulation. To estimate the selection coefficient and selection time, we set the selection coefficient as 0.01, 0.05, 0.07, 0.1, 0.5, and 1 and performed 10,000 simulations for each selection time, ranging from 310 to 1,500 generations, with each step comprising 10 generations. Next, we defined the mean values of the numbers of segregating sites as *S*_*t*_ for generation *t* and the numbers of distinct haplotypes as *H*_*t*_ for generation *t*. S_0_ is the observed number of segregating sites, and *H*_0_ is the observed number of distinct haplotypes. Genetic distance was calculated as follows:


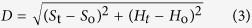


The average distance was calculated as follows:


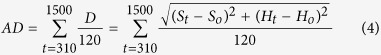


Finally, we chose the selection coefficient with minimum average distance and selection time with minimum distance.

### LD analysis

LD analysis using phase I data from the 1000 Genomes Project was calculated for the CEU, CHB, and YRI populations using the Haploview software (http://www.broadinstitute.org/scientific-community/science/programs/medical-and-population-genetics/haploview/haploview)^28^.

### Recombination rate analysis

Recombination maps were generated from the HapMap phase III^29^ genotype data of three continental populations, i.e., CEU, YRI, and CHB, using the *rhomap* software provided in the LDhat package^30^. A total of 96 unrelated individuals from each population were randomly selected, which is the maximum number of samples that the software can manage.

## Additional Information

**How to cite this article**: Khan, A. *et al.* Genetic diversity and natural selection footprints of the glycine amidinotransferase gene in various human populations. *Sci. Rep.* **6**, 18755; doi: 10.1038/srep18755 (2016).

## Supplementary Material

Supplementary figures

Supplementary Dataset 1

Supplementary Dataset 2

## Figures and Tables

**Table 1:** Haplotype composition of the *GATM* gene in percentage among different populations.

Haplotype name	CEU	FIN	GBR	IBS	TSI	CHB	CHS	JPT	CLM	MXL	PUR	ASW	LWK	YRI
Haplotype-I	61.76	58.6	58.43	60.71	55.1	6.19	8.5	2.25	40.83	33.33	49.09	13.11	5.15	6.25
Haplotype-II	20.59	30.11	23.03	21.43	27.55	81.96	83.00	86.52	40.83	56.82	32.73	20.49	31.44	26.14
Haplotype-III	0	0	0	0	0	0	0	0	5.0	0.76	6.36	43.44	41.75	53.98
Haplotype-IV	16.47	6.45	10.11	14.29	13.78	8.76	7.0	8.99	7.50	3.79	7.27	7.38	7.22	3.41
Other minor haplotypes	1.18	4.84	8.43	3.57	3.57	3.09	1.5	2.25	5.83	5.3	4.55	15.57	14.43	10.23
