# Supplementary material for: Genetic diversity and natural selection footprints of the glycine amidinotransferase gene in various human populations
Source: Sci Rep. 2016 Jan 5;6:18755. doi: 10.1038/srep18755 (PMC4700420; doi:10.1038/srep18755)

Genetic diversity and natural selection footprints of the glycine amidinotransferase gene in various human populations

Running Title: Diversity and Natural selection on *GATM*

Keywords: *GATM, AGAT, Genetic diversity, Natural selection*

Asifullah Khan[1,2,§], Lei Tian [1,§], Chao Zhang [1], Kai Yuan [1], Shuhua Xu[1,3,4,*]

[1]Chinese Academy of Sciences (CAS) Key Laboratory of Computational Biology, Max Planck Independent Research Group on Population Genomics, CAS-MPG Partner Institute for Computational Biology (PICB), Shanghai Institutes for Biological Sciences, Chinese academy of Sciences, Shanghai 200031, China;

[2]Department of Biochemistry, Abdul Wali Khan University Mardan (AWKUM), Mardan, Khyber Pakhthunkhwa, Pakistan;

[3] School of Life Science and Technology, ShanghaiTech University, Shanghai 200031, China;

[4]Collaborative Innovation Center of Genetics and Development, Shanghai 200438, China.

[§]These two authors have contributed equally to this work.

*Correspondence and requests for materials should be addressed to S.X. (xushua@picb.ac.cn).

Supplementary figures legends:

Figure S1: Estimation of pairwise F_{ST} values for the *GATM* gene among different human populations.

Figure S2: Distribution of F_{ST} differentiation ratios for the *GATM* gene and 68 statin response-associated genes. Black dots represent genes that contain no SNPs.

Figure S3: (A) Allele frequency plot of *GATM* eQTL experimentally proved to be associated with statin associated myopathy. (B) Population wide global *Fst* calculation for three eQTLs experimentally proved in association with statin response. Two of these eQTLs (i.e. rs1719247 and rs1346268) are showing allele frequency difference > 1 % whole genome *Fst* differentiation.

Figure S4: Estimation of *lnRsb*, ΔDAF, and F_{ST} around the *GATM* gene. The red and green lines indicate the respective top 1% and top 5% thresholds of these abovementioned parameters at the whole-genome level.

Figure S5: Recombination hotspots observed within the vicinity of the *GATM* gene in the CEU population. The *GATM* gene is indicated in red. Data on the CHB and YRI populations are also shown for comparison.

Figure S6: Linkage disequilibrium (LD) plot across the *GATM* gene in the CEU, CHB, and YRI populations.

Figure S1

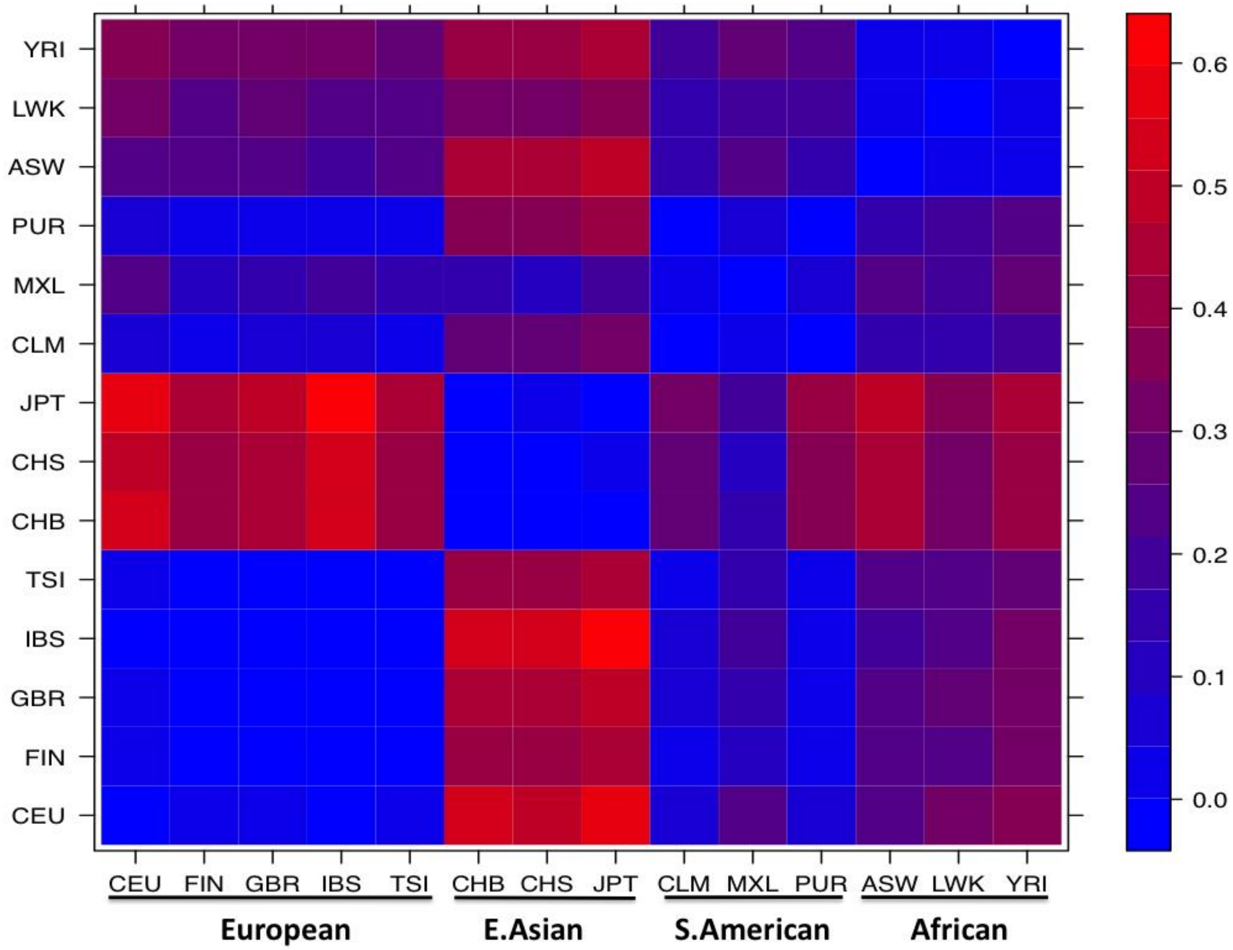

YRI
LWK
ASW
PUR
MXL
CLM
JPT
CHS
CHB
TSI
IBS
GBR
FIN
CEU
CEU FIN GBR IBS TSI CHB CHS JPT CLM MXL PUR ASW LWK YRI
European
E.Asian
S.American
African
0.6
0.5
0.4
0.3
0.2
0.1
0.0

Figure S2

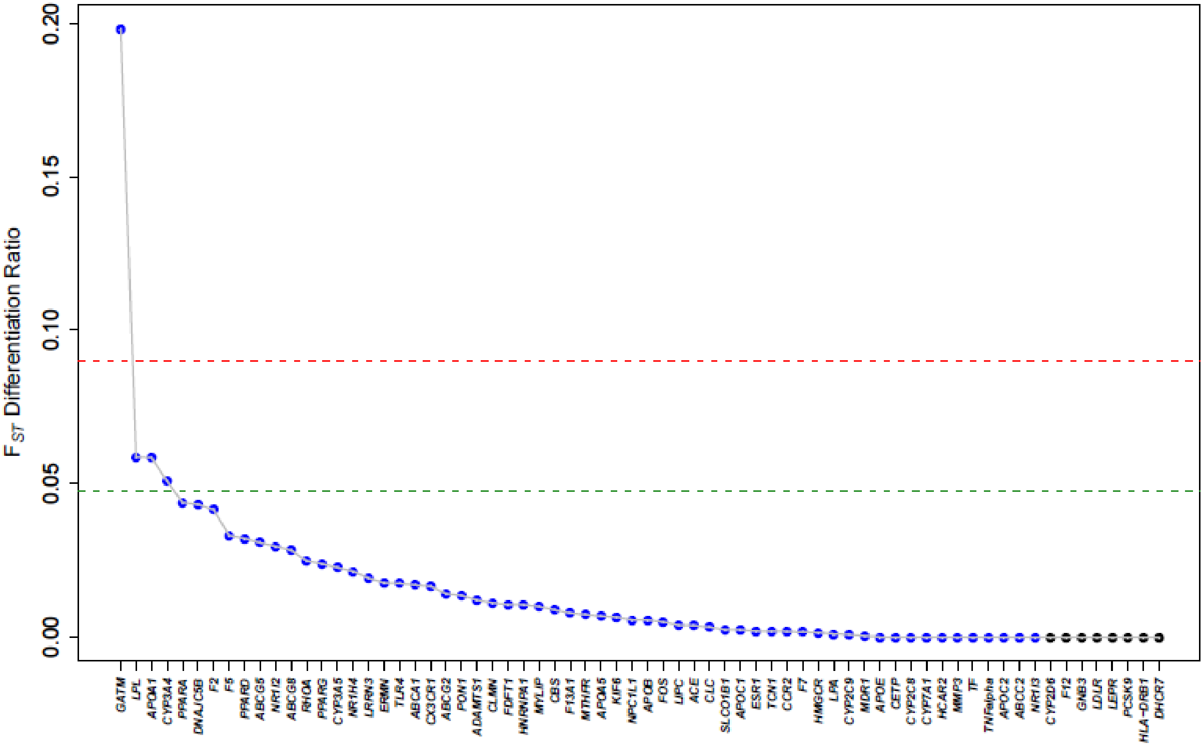

0.20
0.15
0.10
0.05
0.00
F_{ST} Differentiation Ratio
GATM
LPL
APOA1
CYP3A4
PPARA
DNAJC5B
F2
F5
PPARD
ABCG5
NR1I2
ABCG8
RHOA
PPARG
CYP3A5
NR1H4
LRRN3
ERMN
TLR4
ABCA1
CX3CR1
ABCG2
PON1
ADAMTS1
CLMN
FDFT1
HNRNPA1
MYLIP
CBS
F13A1
MTHFR
APOA5
KIF6
NPC1L1
APOB
FOS
LIPC
ACE
CLC
SLCO1B1
APOC1
ESR1
TCN1
CCR2
F7
HMGCR
LPA
CYP2C9
MDR1
APOE
CETP
CYP2C8
CYP7A1
HCAR2
MMP3
TF
TNFalpha
APOC2
ABCC2
NR1I3
CYP2D6
F12
GNB3
LDLR
LEPR
PCSK9
HLA−DRB1
DHCR7

Figure S3

A.

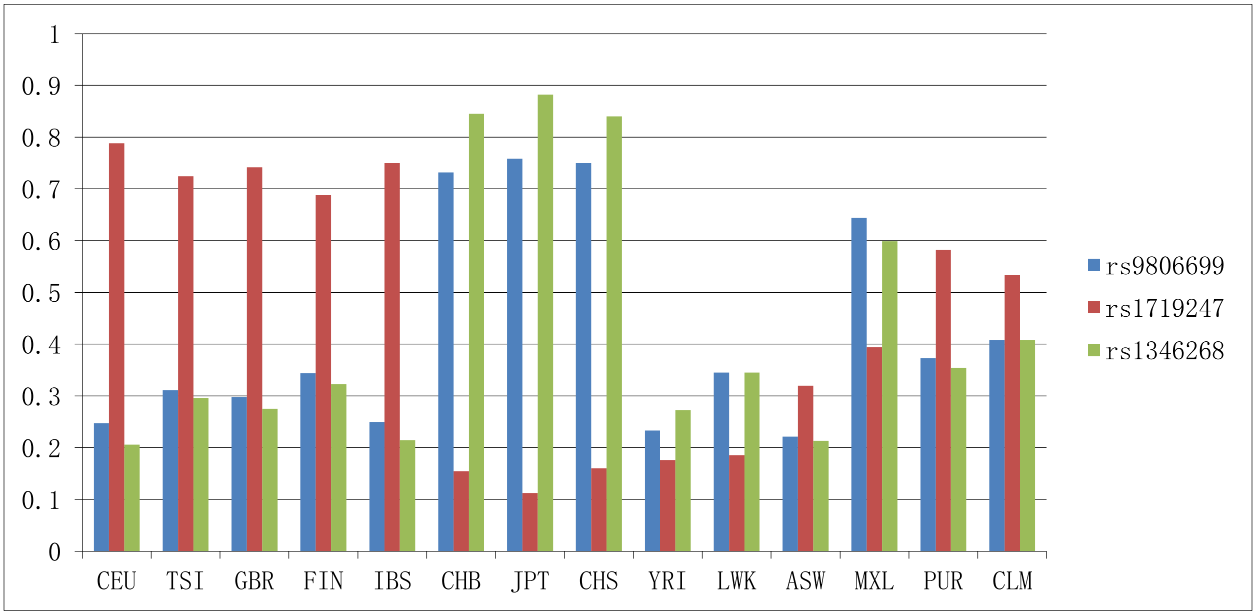
1
0.9
0.8
0.7
0.6
0.5
0.4
0.3
0.2
0.1
0
CEU
TSI
GBR
FIN
IBS
CHB
JPT
CHS
YRI
LWK
ASW
MXL
PUR
CLM
rs9806699
rs1719247
rs1346268

B.

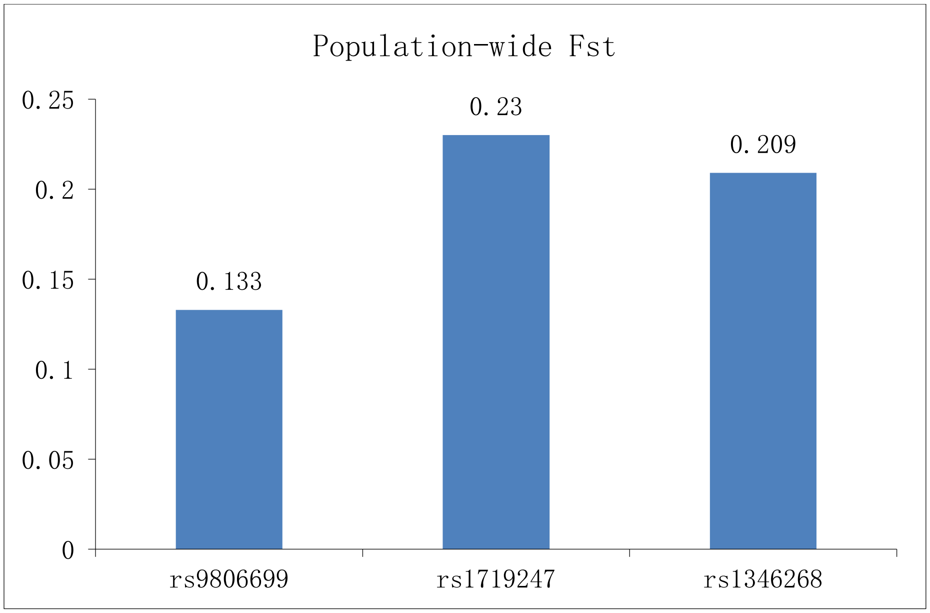
Population-wide Fst
0.25
0.2
0.15
0.1
0.05
0
0.133
0.23
0.209
rs9806699
rs1719247
rs1346268

Figure S4

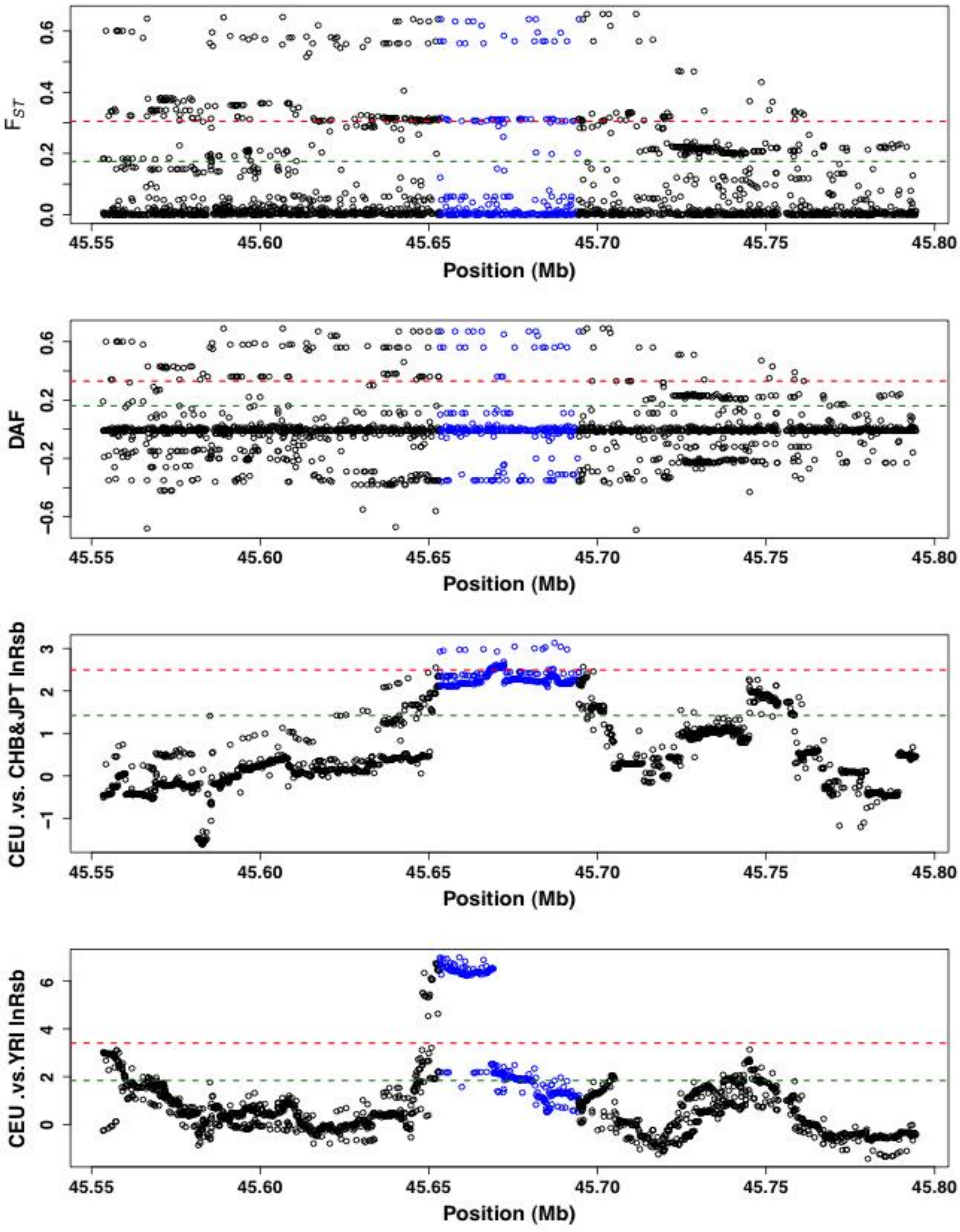

F_ST
0.0
0.2
0.4
0.6
45.55
45.60
45.65
45.70
45.75
45.80
Position (Mb)
DAF
−0.6
−0.2
0.2
0.6
45.55
45.60
45.65
45.70
45.75
45.80
Position (Mb)
CEU .vs. CHB&JPT lnRsb
−1
0
1
2
3
45.55
45.60
45.65
45.70
45.75
45.80
Position (Mb)
CEU .vs.YRI lnRsb
0
2
4
6
45.55
45.60
45.65
45.70
45.75
45.80
Position (Mb)

Figure S5

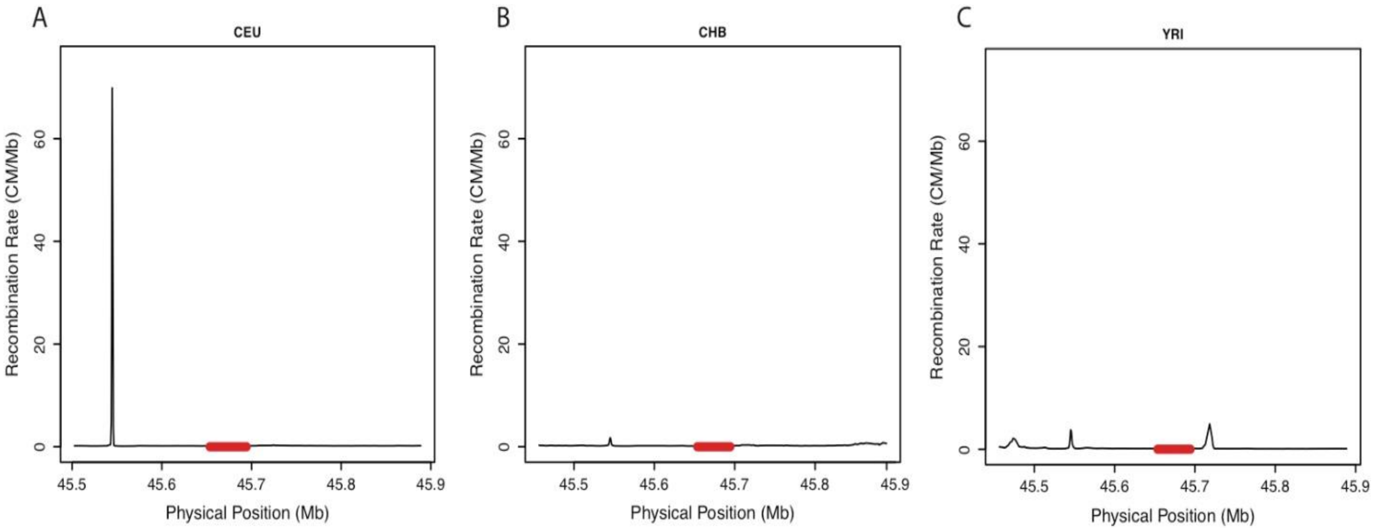
A
CEU
B
CHB
C
YRI
Recombination Rate (CM/Mb)
0
20
40
60
45.5
45.6
45.7
45.8
45.9
Physical Position (Mb)

Figure S6

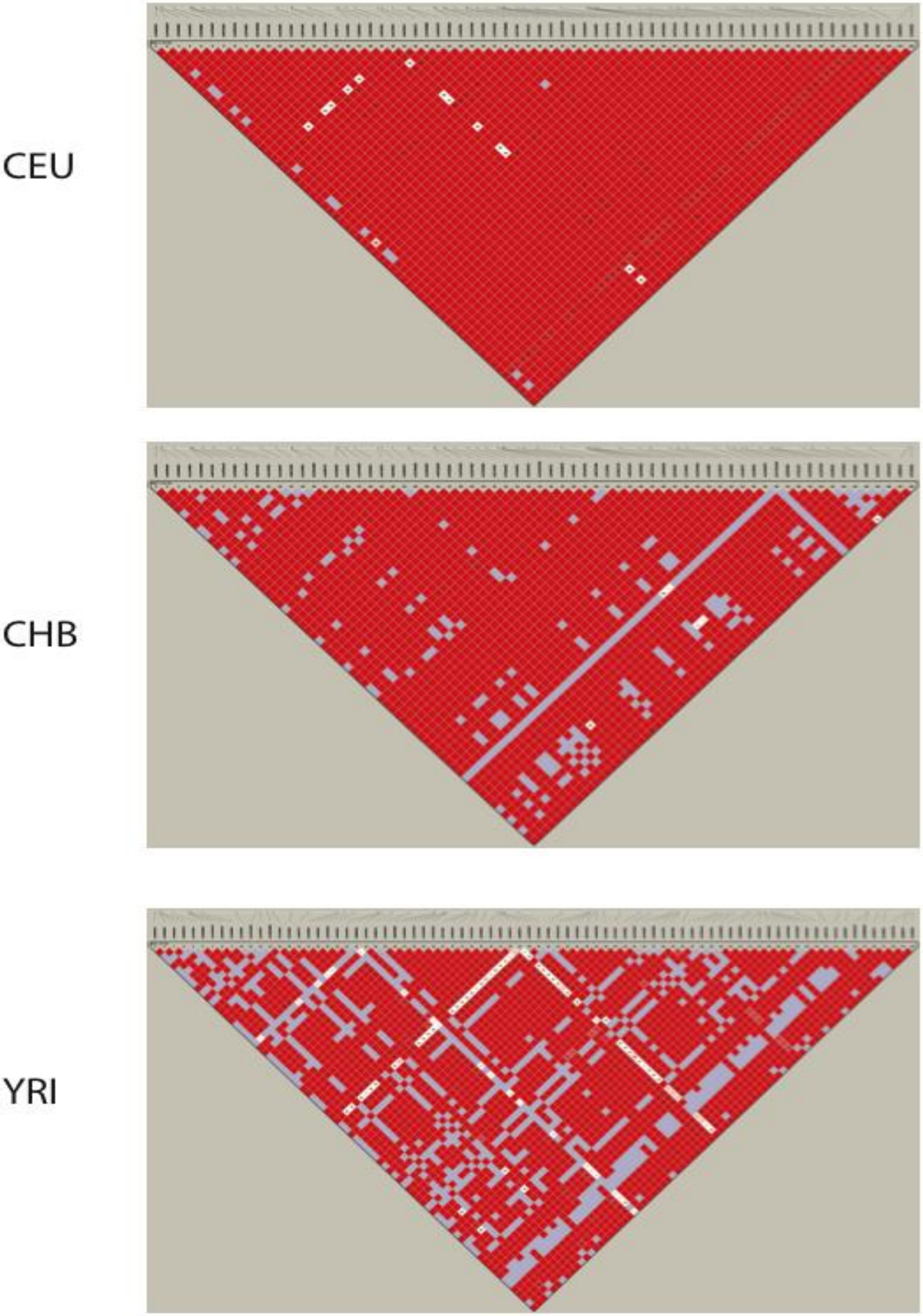

Supplement: Supplementary figures [file srep18755-s1.pdf]
